# Reflections: Improving Medical Students’ Presentation Skills

**DOI:** 10.1007/s13187-016-1005-6

**Published:** 2016-02-26

**Authors:** Radoslaw Tarkowski

**Affiliations:** Department f Oncology, Division of Surgical Oncology, Wroclaw Medical University, pl. Hirszfelda 12, 53-413 Wroclaw, Poland

**Keywords:** Presentation, Lecture, Public speaking skills, Communication

## Abstract

Both good communication and presentation skills on the part of an academic teacher are crucial when trying to generate students’ interest in the subject of a lecture. More generally, our task is to share knowledge in the most effective way possible. It is also worth teaching students presentation skills, as today’s students are tomorrow’s teachers. An engaging presentation is a powerful tool. There are some rules for presenting which I consider worthy of being discussed and taught at a medical university.

Excellent communication and presentation skills are critical when trying to engage students’ interests in the subject of a lecture. Not only should it be important for us to deliver effective lectures, but also we should share our knowledge with students on how to create and deliver effective lectures for today’s students are tomorrow’s teachers. Programs such as Power Point and Keynote have been in use for a long time, and some would argue that everyone knows how to use them and that it is a routine for anyone to prepare an effective presentation and to share their ideas. However, what we often encounter during classes or scientific events are rather boring and ineffective attempts at communication. This can even be seen in the dynamically evolving and thus fascinating field of oncology, where there is always something new to present. Rows of ciphers or dozens of graphs condensed onto one slide together with the monotonous voice of the presenter will tend to discourage even the most attentive listener. Even if the topic of the presentation concerns the milestones of evidence-based medicine, a poor presentation can veil the essentials. What lies at fault is not the color of the presenter’s tie, but more likely the content/format of the slides and the presenter’s delivery. Both of these are easily improved upon thus enhancing the presentation and reception of the lecture.

I changed my way of presenting 4 years ago after being inspired by Garr Reynolds [1] and TED Presentations [https://www.ted.com/topics/presentation]. I have since received much more positive feedback from my audiences. This statement may seem rather subjective and can be read as an unfounded claim, but I am also able to see progress in the results of my work as an academic teacher. Regardless, there are some rules for presenting which I consider worthy of being discussed and taught at a medical university. While our students make occasional presentations during their classes, this activity is often avoided. Students consider the value of their presentations to be lower than those given by their lecturers. This is because students are not taught how to present. We should start to remedy this situation at the undergraduate level in order to make their future professional communication clear, elegant, and intelligent. Since finding time in the general curriculum to teach these skills is impossible, one must find alternative means. This can be successfully achieved with the very active subgroups of medical students such as those who participate in our Student Scientific Society, a volunteer organization of students who want to broaden their knowledge. One of the tasks pursued by this group is how to make effective presentations. Some details concerning this organization have been previously presented in this journal [2].

During regular monthly meetings, students are encouraged to present a chosen topic in a 20-min talk, prepared either individually (Fig. 1) or in small groups (in this case also as an exercise focusing on improving team work). Attention is focused on three areas: appearance, presence, and presentation. Although casual dress is appropriate and acceptable at our informal meetings, those who present at conferences should be dressed more formally as a sign of respect for the audience and their own status as experts (who they will one day become due to their studies). They should appear confident and relaxed. This comes from both the repeated practice of the presentation and being present in the “here and now.” This special kind of presence can be noticed in expert musicians, dancers, sportsmen, zen adepts, or anybody devoted to the mastery of their art, when there is nothing else but the performance [1]. Preparation and knowledge are crucial for minimizing anxiety and jitters, which may manifest in a trembling voice, rapid breath, and finally losing one’s thread and concentration. The voice should be clear, assured, and strong. Students should be instructed in the proper use of a microphone which they will likely encounter in larger teaching venues. For example, the presenter should speak directly into the microphone and if given a choice, it is better to use a portable microphone than to stand behind a lectern which covers the silhouette of the presenter like a bunker, acting as a barrier between the speaker and the audience. The presenter should stand facing the audience, who have come to see him/her and to maintain eye-contact instead of looking at the monitor or looking at the screen with his/her back to the audience. The speaker should talk to the audience and not read from the slides, but to use them as a guide in the presentation (Fig. 2). Students are instructed to limit the amount of information per slide so as to avoid information noise and make the transmission of information clear. Speakers are also encouraged to use pictures to illustrate their topic (“one picture is worth a thousand words”) [1] and tell their story, since this is a story that holds the attention of the audience. Material contained in the slides should be used to illustrate the story, to be a support, a visualization, and guide which does not include everything the presenter says. (The presenter should be aware that the audience reads faster than a lector who reads aloud.) Presentations given by students not familiar with the art of teaching often have far too many slides for the time. Available and they are often disorganized. I instruct students that each slide takes 1 to 2 min and that typical scientific presentations should include the following: title slide; background (one to two slides); problem statement or hypothesis (one slide); methods (one to two slides); results (the number will vary, but should be presented succinctly and as bullet lists, tables, and figures); strengths/limitations (one slide); conclusion and take home message (typically one slide); and acknowledgments (one slide). Students are also advised to incorporate cases as examples. Our participants are told to grasp their audience’s attention, to be engaging and to speak with passion. Although this last skill perhaps cannot be taught, focusing on the present and the idea is very helpful. Of course, if they have joined the Student Scientific Society, this means that they have a passion for their studies, which can be tapped during their presentations. Such training improves the skills necessary to present, discuss, and convince. Students start presenting in front of their peers and then at scientific events.

**Fig. 1:**
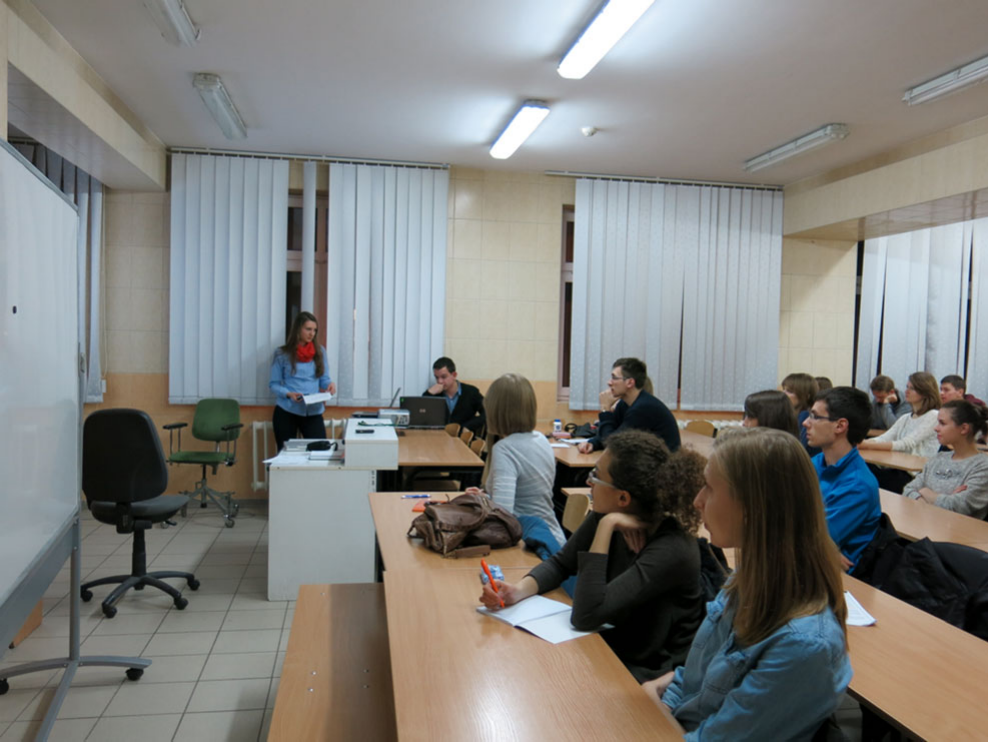
Student presenting

**Fig. 2:**
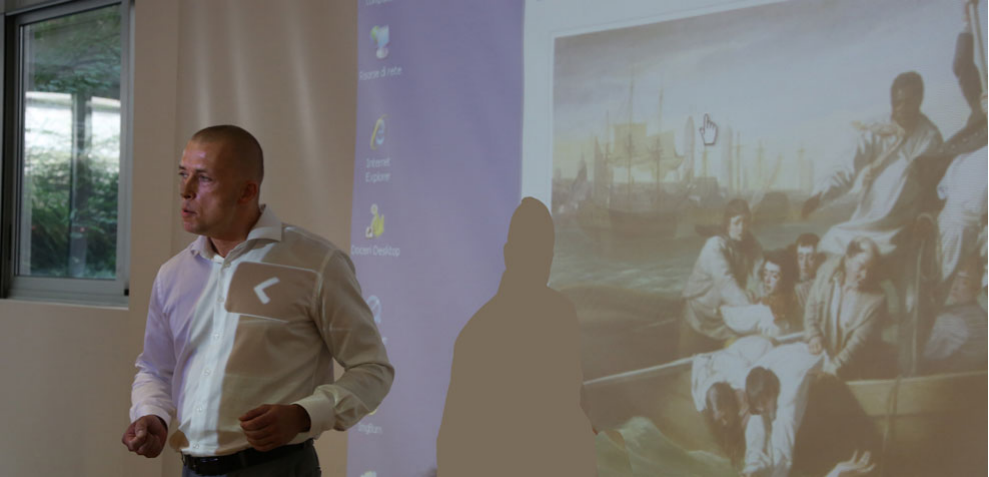
Presenter in front of the audience. Photograph by Peter McIntyre

The number of students actively participating in our Society has increased from 38 in 2011 to 47 in 2012 to 82 in 2013 (I have been a tutor of the Society since 1999). I consider the improvement of my presentation skills to be one of the reasons for this increase in participation by students, as there is a visibly growing proportion of third year students with whom I am in contact. Medical studies in Poland comprise six years and the third year the students follow a module in oncology. This is a time when they can discover the field which interests them. I am convinced that a good presentation is one of the most important elements in attracting their attention.

One of the goals of the Society is to design and conduct scientific studies. Students present their results first at our group meetings and are next presented at scientific events (usually at student conferences) and are sometimes published [3]. Not only is the value of their scientific work deemed important, but also the way that it is presented. This has led to an increased number of prizes: one, 1st prize out of two papers presented by our students in 2011, and three (1st, 2nd, 3rd prize) out of seven presented in 2012, both at international student conferences. I feel very proud watching them present their work at meetings of the European Association for Cancer Education as they have in Wroclaw, Caen, and Heidelberg. Some of the former members of the Society are now oncologists and I am very happy to be able to meet them as friends and colleagues at Tumor Boards or scientific events. I have also discovered the publications of others from our Student Society who have pursued other specializations. I am very proud of our common experience in the education process which has taken us forward together. In conclusion, based on my own observations of student success and their professional growth, I consider that teaching presentation skills is very valuable. These skills should be taught at all medical schools as they help to improve the communication and dissemination of ideas.
